# Preliminary results of the placental decorin profile in bovine pregnancy and parturition

**DOI:** 10.1007/s10719-018-9834-7

**Published:** 2018-07-19

**Authors:** Monika Franczyk, Jacek Wawrzykowski, Marta Kankofer

**Affiliations:** Department of Biochemistry, Faculty of Veterinary Medicine, University of Life Science in Lublin, Akademicka 12, 20-033 Lublin, Poland

**Keywords:** Decorin, Bovine pregnancy, Parturition, ECM proteins, Placenta of cows, Adhesion

## Abstract

Decorin is a small leucine-rich proteoglycan which is involved in multiple biological functions mainly as a structural and signaling molecule. Due to its biological properties in connective tissue, decorin may participate in remodeling of ECM during attachment and detachment of placenta within the course of pregnancy and at parturition in cows. The aim of the study was to detect the presence of decorin protein in bovine placental tissues and to evaluate its profile during pregnancy and at parturition. Placental tissues from healthy pregnant cows (2–5 month) were collected in abattoir (*n* = 10), while parturient tissues were obtained during caesarian section at physiological term (*n* = 6). Maternal and fetal parts were separated manually and subjected to homogenization and to quantitative ELISA and verification by Western blotting with anti-decorin antibodies. ELISA test showed that the concentration of decorin during pregnancy was higher in the fetal part of placenta compared with the maternal part (*p* < 0.001). Similar pattern was noted regarding to maternal and fetal samples derived from parturient cows. Our preliminary results demonstrate that the concentration of decorin is gestation time-dependent in healthy bovine placenta. Possible confirmation of the involvement of decorin in early pregnancy attachment and detachment of the placenta during parturition requires further research.

## Introduction

The regulation of cell-cell and cell-extracellular matrix adhesion might be involved in both appropriate attachment and detachment of placenta during early pregnancy and labour. In most respects, these adhesive processes may include an interplay between extracellular matrix (ECM) proteins. The ECM, which fills the intercellular space and provides an appropriate environment for animal cells and also regulates cell functions through signaling, proliferation, migration and invasion, consists of several simple proteins and proteoglycans [1].

During early pregnancy and periparturient period, characteristic alterations related to the amount and the distribution of ECM components, such as: collagens, fibronectin, laminin and integrins are noted [2]. Type-specific and location-specific distribution patterns of collagens within the uterus of pregnant cows reflect the dynamic processes at the stromal level [3].

Notwithstanding, apart from these structural proteins there are several biologically active proteins, which are conjugated with sugar moieties. One of representatives is decorin (DCN; UniProtKB/Swiss-Prot: P21793; MW of protein core: 39.879 Da; length: 360 aa; pI: 8.72), which is a small leucine-rich proteoglycan first time isolated from bovine placenta in 2000 using chromatographic methods [4]. As a result of post-translational modifications of the decorin protein, a proteoglycan is formed, which molecular mass changes depending on attached glycosaminoglycan: chondroitin 4-sulfate, chondroitin 6-sulfate or dermatan sulfate (UniProtKB/Swiss-Prot). DCN is involved in multiple cellular functions such as proliferation, migration and invasion and acts as a structural molecule, as well as a ligand for receptors [5, 6].

The main function of decorin seems to be the involvement in ECM organization, including the interaction and regulation of collagen self-assembly. DCN is involved the formation of collagen fibers during fibrillogenesis causing changes in its properties [7]. The highest binding affinity of bovine decorin has been observed to type III, I and V collagen respectively [4].

What is more, the characteristic feature of decorin is its anti-adhesive capacity, what has been confirmed in many experiments, mainly based on human cells. One of the assumptions concerning adhesion is that the presence of decorin in ECM regulates the formation of collagen fibers leading to normal adhesion.

Little is known about the role of decorin in the periparturient period in cows. Available sources of information report that changes in the expression of decorin may be associated with the maturation of the placenta and pregnancy development [8], thus it appeared to be a candidate protein for regulation of the attachment and detachment of the placenta.

In the study of human fetal membranes the amount of both decorin and collagen decreases prior to parturition as membrane tensile strength declines [9, 10].

Since decorin exhibits a wide range of functions during the ECM remodeling, it aroused the interest of researchers in pathology. Some reports demonstrate that there is a difference between patterns of decorin taking into account physiological and disturbed placentas in humans [11].

In our study we focus on the investigation of decorin protein concentration in healthy placentas derived from early pregnancy and at term.

## Materials and methods

### Tissue collection and homogenization

The uteri of slaughtered cows were immediately visually examined for evidence of early-mid pregnancies. Health status was confirmed in sanitary examination and by additional examination of uterus and fetus. Although in accordance with law it is not allowed to slaughter pregnant animals such cases can be noticed and in fact they give good opportunity to examine pregnancy pathway.

Placental samples from pregnant healthy cows (*n* = 10) with a gestational age of 2–5 months (3 × 2 months, 5 × 3 months, 1 × 4 months, 1 × 5 months) were obtained from the local abattoir, immediately after slaughter. Fetal age was estimated by measurement of the crown-crump length of the fetus (8-37 cm). The approval of the ethics committee was not required, since biological material was collected postmortem under approved procedures.

Placental samples at the time of parturition were collected from healthy cows (*n* = 6) admitted for routine caesarian section to veterinary clinic. Placentomes were excised always from similar location in uterus.

Placentomes were separated manually into maternal caruncles and fetal villi manually and then frozen and stored at −20 °C until used. Always similar fragment of placentome was taken for further analysis.

Placental maternal and fetal tissues were washed in cold 0.9% NaCl and homogenized (4 °C, 5 min, at the speed 10,000 rev/min) in phosphate buffer (0.05 M at pH 7.2) with addition of TRITON X-100 and in the presence of protease inhibitor cocktail (P2714 SIGMA; containing: AEBSF Aprotinin Bestatin E-64 EDTA, Leupeptin) using Ultra Turrax T 25 (Ikawerk Janke and Kunkel Inc., Staufen, Germany). After centrifugation (4 °C, 20 min, 6000 × g) supernatants were collected and analyzed for total protein, Western blot and ELISA.

### Total protein determination

The total protein concentration of homogenates was measured based on the biuret reaction as described in [12] using commercial available kit (Cormay, Lublin, Poland). The measurement of the absorbance of the samples was done in duplicate at a wavelength of 546 nm.

### Western blotting

A total of 30 μg of protein from each tissue homogenate was subjected to 1D 12.5% SDS-PAGE using Mini PROTEAN® Tetra Cell (Bio-Rad, Warsaw, Poland). Samples derived from each cow were assayed in duplicate. The running buffer was 0.025 M Tris/glycine at pH 8.3. The samples were mixed with a sample buffer with reducing agent. Protein separation was performed with 1.5 mm thickness 8.2 × 7 cm gels and conducted at constant voltage of 100 V. Obtained protein fractions were transferred onto Immun-Blot PVDF membranes (2 h, 100 V at 4 °C) with Criterion BLOTTER (Bio-Rad, Warsaw, Poland) and incubated for 1 h in 4% solution of non-fat milk to eliminate non-specific immune response. For decorin detection, specific immune reaction was performed with 1 μg/ml sheep polyclonal anti-Decorin antibodies (RayBiotech, Norcross, Georgia) for 12 h at 4 °C. The visualization of immune reactions was prepared as previously described [13] with secondary polyclonal donkey antibody to sheep IgG conjugated with alkaline phosphatase at a concentration of 0.25 μg/ml. The obtained bands were compared with the decorin standard (RayBiotech, Norcross, Georgia). Dark fractions located at expected molecular weight positions were considered as positive immune reactions. As negative control, primary antibody was omitted and resulted in the lack of dark fractions at expected location. The membranes were scanned and the molecular weight and relative quantities were estimated with the Bio-Rad Quantity One 4.1 Software.

### Elisa

For ELISA analysis the Bovine Decorin ELISA Kit, (ELB-Decorin, RayBiotech, Norcross, Georgia) was used according to manufacturer’s instructions. All steps of the procedure were conducted at room temperature. Samples derived from each cow were analyzed in duplicate. 100 μl of tissue homogenates was added to pre-coated 96-well microplate and incubated 2.5 h. After 4× washing steps, plate was then incubated 1 h (shaking) with biotinylated antibody. After rinsing unbound antibodies, 100 μl of streptavidin-conjugated HRP solution was added to each well and incubated for 45 min. For colour development wells were incubated in 100 μl of TMB Substrate for 30 min and then 50 μl of H_2_SO_4_ was added to stop the reaction. Measurement of the absorbance at 450 nm was done immediately on microplate reader (Labsystems Multiskan RC) and standard curve and calculated data values were performed using Genesis software (GENESIS LITE, Version 3.03, Life Sciences, UK). Outcomes were expressed as pg of decorin per mg of total protein (pg/mg).

### Statistical analysis

All obtained data was analyzed using IBM SPSS Statistics software. Comparisons of decorin concentrations between groups (pregnancy vs. parturition, separately for the maternal and fetal parts) were performed with analysis of nonparametric Mann-Whitney U-test. The probability value of <0.05 was considered statistically significant. Data are presented as means ± SD.

## Results

**Fig. 1:**
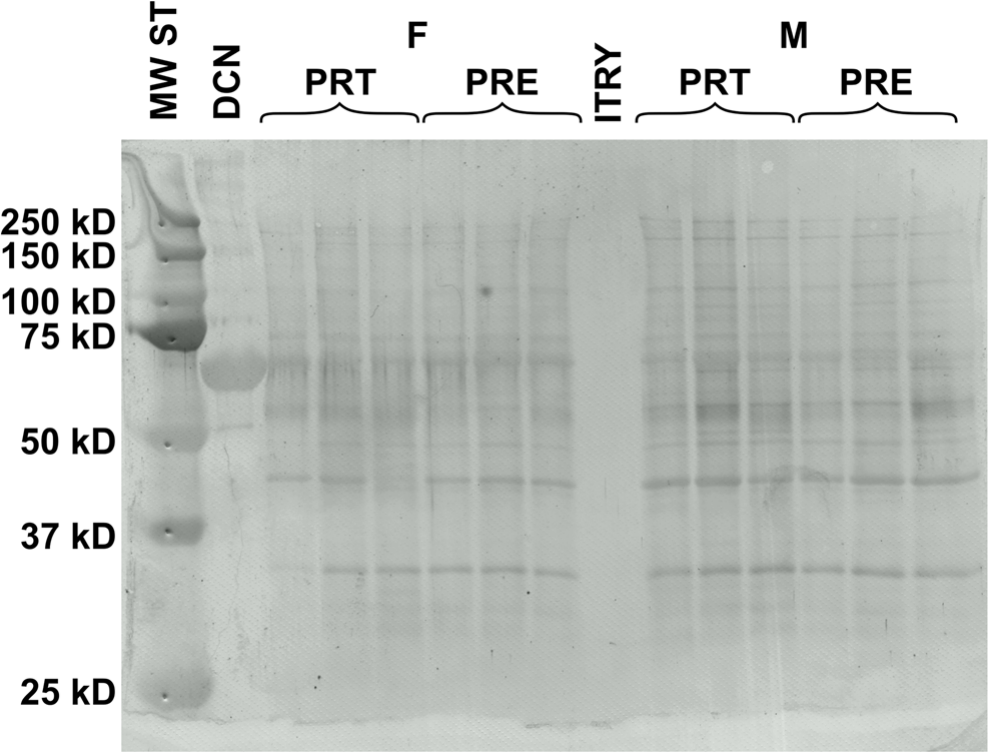
Results of Western blotting analysis from bovine placental samples demonstrate the presence of decorin (approx. 65 kDa) during pregnancy (PRE) and parturition (PRT) in fetal (F) and maternal (M) part of the placenta. MW ST – Molecular mass of protein standard (Precision Plus Protein™ Dual Color Standards, Bio-Rad); DCN – Decorin standard (RayBiotech, Norcross, Georgia); ITRY – Trypsin inhibitor soybean as a negative control (1,856,274, Thermo Scientific)

Western blotting confirmed the presence of decorin both in maternal and fetal part of bovine placenta during pregnancy and at delivery (Fig. 1). Placental homogenates exhibited DCN protein with molecular mass around 65 kDa. Interestingly, within the fetal side there was a single band of protein representing decorin, whereas multiple bands were apparent on the maternal side. In accordance with available literature it might indicate a potential post-translational modifications.

**Fig. 2:**
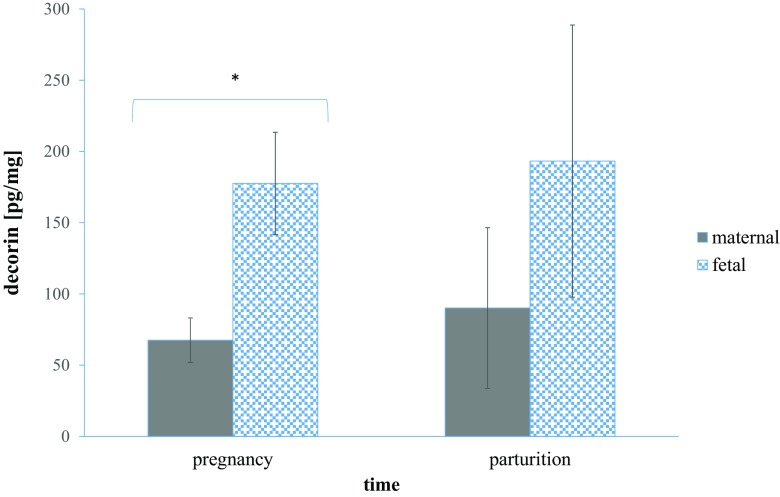
Concentrations of decorin (pg/mg protein) within pregnancy and parturient period in cows analyzed by determination of the absorbance at 450 nm. Data are derived from the experiment performed in duplicate (* *p* < 0.001). *p* value was calculated based on Mann-Whitney U-test

The concentration of decorin protein was detected in placental samples by using ELISA method. The results showed dynamic alterations taking into account the distribution of DCN within maternal and fetal part (Fig. 2). Decorin levels during pregnancy were higher in the fetal part of placenta comparing with maternal part (177.54 ± 35.95 pg/mg protein vs. 67.54 ± 15.64 pg/mg protein, respectively; *p* < 0.001). Similar dependence was observed in the case of parturient cows (193.26 ± 95.50 pg/mg protein - for fetal part, and 90.13 ± 56.36 50 pg/mg protein - for maternal part), however these results were not statistically significant. Temporal patterns of DCN concentration within examined time points were comparable, however there was a slight increase during parturition compared to pregnancy (90.13 ± 56.36 vs. 67.54 ± 15.64 pg/mg protein – for maternal tissue, and 193.26 ± 95.50 vs. 177.54 ± 35.95 pg/mg protein – for fetal tissue). The lack of statistical significance is due to the fact that samples showed high deviation.

## Discussion

In our study we focused on decorin, a small proteoglycan of ECM, and revealed for the first time its concentration in the placenta of healthy cows in periparturient period. The presence and quantification of DCN was examined using Western blot and quantitative ELISA.

There is little information in the literature about the changes in the protein profile of bovine placenta during early pregnancy including placentation due to difficulties with access to the biological material. The main reason is limited number of pregnant cows intended for slaughter and difficulties in collecting samples from live animals. Knowledge of this topic is very important for better understanding feto-maternal dependencies throughout gestation and during delivery and should be deepened.

Available publications focused on the study of decorin mRNA expression, but little is known about the exact amount of decorin protein in the bovine placenta – its distribution and alterations during pregnancy and at parturition. As mRNA level may not usually predict its protein level, amount of mRNA cannot be a suitable substitute for the corresponding protein amount [14], consequently protein profile should be equally analyzed.

Besides primary role of decorin in collagen assembly, it is also recognized as an antiadhesive protein and anticoagulant. It may bind to heparin cofactor II and interact with various angiogenic growth factors, including EGF receptor and vascular endothelial growth factor. In the research on human microvascular endothelial cells, reduction of DCN expression resulted in declined proliferation and vessel formation [5].

DCN decreases the number of human adherent endothelial cells [15] and inhibits the function of VEGFR in human placenta exhibiting anti-adhesive properties in the attachment of human trophoblast cells [16].

Interestingly, the presence of decorin protein without sugar moieties also leads to increased adhesion by thickening collagen proving that the properties of DCN depend on its glycosylation [6].

Moreover, decorin affects the movement of cells via the modulation of interaction between receptors on the membrane surface and matrix protein ligands. Cells which expressed decorin have lower ability to migration and besides it, they also accumulated matrix fibronectin [17].

There is an evidence that the expression of decorin in cows shows meaningful changes during placentation. In accordance with Guillomot *et al*., before the attaching of the embryo to the bovine uterine wall, DCN is expressed merely in the stromal cells of the caruncle. In the further development of the embryo during the early stage of placentome generation, decorin mRNA expression increases in the endometrial stromal cells, thus the decrease occurs in the caruncular apical stroma. In turn, with reference to the chorionic tissue DCN mRNA expression declines within the villi after the first trimester. Decorin protein is absent in the whole endometrial stroma and in the mesenchyme of the growing secondary villi of the placentome, which indicates that the nucleic acid and protein profiles differ from each other in the course of pregnancy. Guillomot *et al*. stated, that the deficiency of DCN at early stages of placentation is associated with propagation and migration of the cells, that are involved in this process. Authors confirmed time related changes in DCN profile within maternal and fetal bovine placenta what is in agreement with our results [8].

Riding *et al*. [18] performed proteomic analysis of bovine amniotic and allantoic fluids collected during early gestation (45th day) and thereby identified other ECM proteins, such as: procollagen types I (alpha1, alpha2), III (alpha 1), V (alpha 1) and XVIII (alpha 1) isoform 1 precursor; fibulin 2 precursor; fibronectin 1 and similar to fibronectin 1 isoform 7 preprotein. The presence of these proteins in conceptus fluids may indicate that they are substantial during early stages of gestation.

The outcomes of the present study demonstrate time related changes in the amount of decorin during the course of pregnancy and at parturition in healthy cows. Decorin protein was present in the placentomes derived from pregnancy as well as in the parturient tissues. As shown by Western blotting, within the maternal side there was a fraction representing decorin, which was not detected in the fetal part. This result is probably associated with changed post-translational processing. Since glycosylation modifies the cell behavior [6, 19], we can hypothesize, based on available literature, that this alteration may result in different cell properties within feto-maternal interface.

The present data show, that concentration of decorin had a tendency to be higher at parturition than in placenta of pregnant cows. A slight increase in DCN concentration within examined time points, was not statistically significant due to high deviation of results.

In addition, alterations in the concentration of decorin within maternal and fetal part during the placentation period and fetal development were observed. The amount of DCN within placental fetal tissue was more than 2-fold higher than in maternal part. Different pattern of decorin may indicate specific role within placental cells derived from maternal and fetal parts. Reduced amount of decorin in caruncle may be associated with stronger adhesion properties, enabling maintenance of the placenta during pregnancy. Thus, increase in its amount at parturition, although not statistically significant, may be a signal to expel the placenta during delivery.

Available literature provides with only scarce data on decorin concentration in plasma. Gubbiotti *et al*. [20] provided with the evidence that Decorin is also a secreted proteoglycan found in the blood of non pregnant mice. The average of plasma decorin levels in mice ranges between 40 and 80 ng/ml and it is induced by nutrient deprivation *in vivo*. Moreover, in esophageal squamous cell carcinoma in humans decreased plasma decorin concentrations (5.6 ± 3.6 ng/ml) were observed compared to control patients (7.8 ± 3.1 ng/ml) [21].

Our preliminary results reveal time related changes in decorin in bovine maternal and fetal placenta within physiological pregnancy. Taken together, we suggest, that decorin may be involved in the attachment during early pregnancy and detachment of placenta during parturition, however confirmation of this assumption requires further more detailed research.
